# Using Pulses in Baked Products: Lights, Shadows, and Potential Solutions

**DOI:** 10.3390/foods8100451

**Published:** 2019-10-02

**Authors:** Andrea Bresciani, Alessandra Marti

**Affiliations:** Department of Food, Environmental, and Nutritional Sciences, Università degli Studi di Milano, Via G. Celoria 2, 20133 Milan, Italy; andrea.bresciani@unimi.it

**Keywords:** pulses, bread, bio-technological processing, air classification, fermentation, germination

## Abstract

Nowadays, consumers are more conscious of the environmental and nutritional benefits of foods. Pulses—thanks to both nutritional and health-promoting features, together with their low environmental impact—satisfy the demand for high-protein/high-fiber products. However, their consumption is still somewhat limited in Western countries, due to the presence of antinutrient compounds including phytic acid, trypsin inhibitors, and some undigested oligosaccharides, which are responsible for digestive discomfort. Another limitation of eating pulses regularly is their relatively long preparation time. One way to increase the consumption of pulses is to use them as an ingredient in food formulations, such as bread and other baked products. However, some sensory and technological issues limit the use of pulses on an industrial scale; consequently, they require special attention when combined with cereal-based products. Developing formulations and/or processes to improve pulse quality is necessary to enhance their incorporation into baked products. In this context, this study provides an overview of strengths and weaknesses of pulse-enriched baked products focusing on the various strategies—such as the choice of suitable ingredients or (bio)-technological approaches—that counteract the negative effects of including pulses in baked goods.

## 1. Introduction

Legumes or pulses are dry edible seeds of plants belonging to the Fabaceae (*Leguminoseae*) family, which include field peas, dry beans, lentils, chickpeas and faba beans. The contemporary definition of pulses excludes oilseed legumes and legumes consumed in immature form [1]. Egypt and India consume the largest quantity of pulses; in these regions, pulses play a key role in helping the population to consume suitable levels of several important nutrients, particularly proteins, while in developed countries protein intake is mainly due to the consumption of animal-derived proteins [2]. In Europe, 60% of pulses are consumed in Spain, France, and the UK. It is also important to consider that the way pulses are prepared varies depending on world regions [3]. Nonetheless, pulses are traditionally consumed whole or split after soaking and cooking, although recently they have become increasingly popular and are widely used in food products such as pasta, bread, and other bakery products [4]. Indeed, legumes or pulses represent one of the possible ways to help solve global food security challenges. Indeed, as an inexpensive, sustainable source of proteins and other key nutrients, pulses meet the nutrition and food security requirements of the global population and can support the creation of sustainable and stable agricultural production systems, which could limit the negative effects due to climate change. In this context, the year 2016 was designated by the United Nations as the International Year of Pulses. The purpose of this initiative was to increase public awareness of the nutritional benefits of pulses and their potential role in improving global food security. Three years later, the impact of the International Year of Pulses is still making itself felt.

Pulses are mainly consumed as a whole, but Western countries are increasingly using pulse flour in food preparations for the general population or followers of special diets such as vegetarian, vegan or gluten-free. However, the consumption of pulses is limited due to the presence of antinutrients such as phytic acid, trypsin inhibitors and some non-digestible oligosaccharides that are related, for example, to digestive discomfort [5]. Moreover, the presence of off-flavors discourages the consumption of pulses for some [6]. Therefore, if we want more people to enjoy the scientifically recognized nutritional and health benefits of pulses, it is necessary to find ways and means of improving their quality as ingredients in baked products. In this frame, this review presents:

- the agronomic, compositional, and nutritional benefits of pulses;
- the pro and cons of using pulses in baked products;
- the various approaches for counteracting the negative effects of including them in food formulations.

## 2. Agronomic, Compositional, and Nutritional Benefits

**Figure 1:**
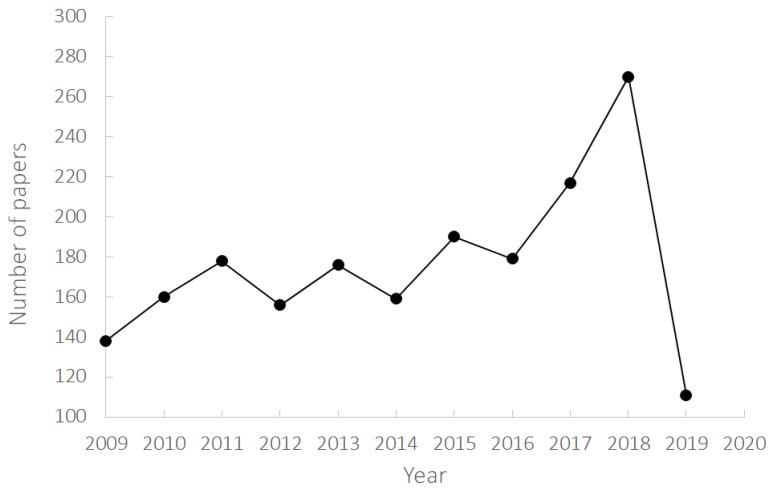
Papers on legumes in the field of food science and technology (source: Web of Science; 2009–2019; updated to 4 July 2019).

The awareness and demand for pulses is still growing, and new pulse-containing products are launched on the market every year to meet the demand for products that are gluten-free, high in proteins and fiber, with a low glycemic index and a clean label. Moreover, end-use applications of pulses have generated research interest in many disciplines, such as breeding, genetics, agronomics, health and nutrition. An overview of the scientific literature of the last ten years setting ‘legumes’ or ‘pulses’ as a search term, resulted in the identification of about 1934 scientific papers in the area of Food Science and Technology (Figure 1). The graph in Figure 1 highlights that the number of publications has constantly been increasing over the years (except for 2019, which is still in progress), suggesting that the interest in this topic is still growing. Moreover, the marked increase in publications since the 2016 should be noted, possibly because of Year of Pulses announcement by FAO. 

Taking into consideration all the pertinent research disciplines, more than 700 review papers have been published on pulses in the last five years. A tentative classification of the reviews published in the last two years according to their particular research area is summarized in Table 1; most concern plant science and agronomy (43%), while others (15%) are dedicated to the development of food products, including bread, pasta, snacks and cookies, enriched with pulses to improve their nutritional properties. Finally, the nutritional properties and the health benefits of pulses are the focus of about 10% of the total reviews.

### 2.1. Agronomic Traits

From an agronomic standpoint, pulses contribute nitrogen to the soil rather than extracting it from the soil. Many of them (e.g., lentils, field peas, and chickpeas) are also more drought- and temperature-tolerant than corn or soybeans. In addition, they tend to be disease-resistant and grow well in areas where weed pressure is low, thereby minimizing the need for pesticides and herbicides [34]. Pulses play an important role in improving soil fertility and would be of great importance for farmers with no or limited access to nitrogen fertilizers [35]. Cultivation of pulses and their use in food formulations instead of cereals could be an ideal approach to reduce and control the effects of climate change. In fact, pulse cultivation has low environmental impact, thanks to their low carbon and water footprints. Carbon footprints are mainly associated with agricultural greenhouse gas emissions [36]. The water footprint of pulses has much less impact compared to that of cereals and other protein sources, such as milk, chicken eggs and meat [37]. Their higher sustainability compared to cereals makes pulses appealing ingredients for food production. 

### 2.2. Compositional Traits

Pulses have a different chemical composition compared to cereals: they are lower in carbohydrates (60–65%) but richer in proteins (21–25%) and fibers (12–20%), and for this reason, pulses are a suitable ingredient for the reformulation and enrichment of bread [5]. 

The primary storage carbohydrate of pulses is starch, which constitutes a major fraction of total carbohydrates for almost all the species [38]. Starch is composed of amylose and amylopectin. The ratio between these components depends on the starch structure. Generally, in cereals the quantity of amylose is about 18% even if values up to 30% have been observed in high amylose varieties (mainly rice and corn) [39]. Pulse starch provides health benefits because its high amylose content promotes the formation of resistant starch that cannot be hydrolyzed during digestion. Also, dietary fiber remains undigested in the small intestine, whereas it is fermented by the microbiota in the colon. Colonic fermentation leads to the growth of beneficial bacteria and an increase in the production of short chain fatty acids, which have been associated with reduced risk of colon cancer [40]. High amylose content makes the starch structure more compact and limits its gelatinization capacity during heating. Moreover, starch remains partially crystalline during cooking due to its cell walls which remain undisrupted during heating. The preservation of the cell walls in whole pulses after cooking seems to prevent starch hydrolysis by digestive enzymes. This may contribute to both the high levels of resistant starch and low glycemic index of pulses [41]. 

As regards non-starch polysaccharides, pulses are a significant source of dietary fiber. There is a wide degree of variation in the amounts of dietary fiber as well as the ratio of soluble to insoluble fiber in pulses [42]. The total dietary fiber content in pulses range from 14 to 32% (dry weight) depending on the species [41]. There are various types of dietary fiber in pulses, including long chain soluble and insoluble polysaccharides, galacto-oligosaccharides and, as mentioned above, resistant starch. While insoluble fiber is generally combined with laxation, soluble fiber is linked with reducing cholesterol levels and ameliorating post-prandial blood glucose levels. Both soluble and insoluble fibers can act as prebiotics, supplying nutrients for gut microorganisms. Flours and fiber-rich fractions from pulses can be successfully utilized to increase the dietary fiber content (soluble and insoluble fiber) of processed foods, which has been shown to have health benefits. As for nutritional and health-promoting effects, pulse fibers can also be useful to improve the textural properties of foods by binding and retaining fat and/or moisture [42].

As regards sugars, monosaccharides generally make up less than 1% of pulse seed weight, whereas oligosaccharides make up 14%. In contrast to monosaccharides, oligosaccharides (i.e., raffinose and stachyose) are non-digestible by humans because of the β-glycosidic bond that links monosaccharides together and these oligosaccharides pass undigested through the stomach and upper intestine. In the lower intestine, they are fermented by gas-producing bacteria and make carbon dioxide, methane, and/or hydrogen, leading to the flatulence commonly associated with eating pulses [5]. 

Pulses contain relatively high amounts of proteins—about twice as much proteins as cereal grains—and for this reason, in many regions of the world, pulses are the major source of dietary protein and often represent a supplement for other protein sources. The most abundant storage proteins in pulses are globulins and albumins, which are classified as soluble proteins. Globulins (soluble in salt-water solutions) represent approximately 70% of the total proteins in pulses, whereas albumins (soluble in water) account for 10–20% [43]. Pulse proteins have low levels of sulfur amino acids, but the amount of lysine is greater than cereals. Therefore, pulse and cereal proteins are nutritionally complementary [44]. In addition to these nutritional features, pulse proteins are interesting from a technological standpoint due to their functional properties, including solubility, water holding capacity, and emulsifying and foaming properties that have been extensively described by several authors [30,31,32,33] as reported in Table 1. 

Pulses are also rich sources of many micronutrients, including selenium, thiamin, niacin, folate, riboflavin, pyridoxine, potassium, zinc, vitamin E, and vitamin A [5]. In addition to being a good source of fiber, proteins, and micronutrients pulses are a source of compounds with antioxidant activity—i.e., flavonoid compounds, such as anthocyanins, quercetin glycosides, and proanthocyanidins, and isoflavones—that could limit the risk of certain diseases and promote overall health [45]. 

### 2.3. Potential Health Benefits

Consumption of pulses is encouraged in the diets of the general population, as their nutrient profile can have a positive health impact. Evidence of the link between the nutritional composition of pulses and the reduction in the risk of cardiovascular disease (CVD) and diabetes comes from more than 2000 studies published in the last ten years (source: Web of Science; updated to 09 September 2019). The potential health benefits of the consumption of beans [46], chickpeas [47], lentils [48], and peas [49] are summarized in Table 2. The intent of the authors was to provide a summary of the health benefits of pulses, not an extensive review. For more details about the nutritional and health benefits of pulses, we refer to the reading of recent reviews [5,50,51,52]. In collecting the information from the above-mentioned papers, the authors have noted that the majority of the studies focused on grains, while the health benefits of pulses-enriched baked goods have not been systematically evaluated, suggesting further studies to fill the current knowledge gap in this research area. 

The high fiber and protein content of pulses has been shown to result in an increase in satiety and may contribute to decreasing the occurrence of obesity by reducing calorie intake and managing body weight over time [53]. These aspects have been shown to decrease the risk of developing type 2 diabetes and CVD [54].

Also, lipid profiles—sterols and mono-and polyunsaturated fats—contribute to reduce overall risk of CVD and atherosclerosis, decreasing total serum triglycerides and cholesterol [55].

Pulses are an ideal food choice for individuals with diabetes thanks to their low GI and high fiber content. Consuming pulses as part of a low-GI diet improved glycemic control, may help lower the risk of diabetes-related complications [56]. Additionally, insulin sensitivity and glucose tolerance are also improved with the presence of resistant starch that is high in pulses, which also helps reduce risks associated with diabetes. Therefore, it has been shown that the consumption of pulses is beneficial to the management of type 2 diabetes, metabolic syndrome and obesity. Pulse consumption is also linked to a reduction in cardiovascular disease and risk of cancer, they also contribute to overall health and wellness [57]. Moreover, the absence of gluten in pulses allows celiac disease sufferers to digest them. 

The agronomic, compositional and nutritional benefits of pulses are the driving force for the growing interest in producing pulse-derived foods that are healthy, convenient, and rich in protein and fiber. In this context, they are usually processed to obtain flour or fiber, starch, and protein concentrates or isolates [59]. These different ingredients could be used in food reformulation to improve the physico-chemical, nutritional and technological properties of baked products. 

## 3. Using Pulses in Baked Products

The growing interest in gluten-free, vegan and vegetarian diets has resulted in an increase in pulse consumption. Flour from pulses is mixed with other grains (with or without gluten) to make bread, biscuits or cookies, and other baked products. The following section will summarize the strengths and weaknesses of pulse-enriched foods in the past ten years.

### 3.1. Bread

Bread, a traditional and economical product that is easy to prepare and consume, is one of the most popular foods worldwide and is generally prepared from common wheat. Thus, it is a source of calories and of complex carbohydrates, with a modest amount of essential amino acids such as lysine and threonine. Using refined white flour instead of wholemeal flour, however, reduces the nutritional density and fiber content of white bread [60]. Nowadays consumers are more health oriented and conscious of the environmental and nutritional benefits of food. In response to consumer demands, the food industry is formulating vegetable-based products that fully satisfy the health and cultural concerns of today’s typical consumer. From this point of view, pulses are a potential ingredient to improve the quality of products that are already widely consumed. Pulse-enriched wheat flour represents a potential way to increase the nutritional properties of cereal-based foods; it is well known that the amino acid composition of pulses complements that of cereals [43]. They are also rich in bioactive compounds, including fiber [61]. In addition, pulses are characterized by reduced starch bioavailability and high resistant starch content. Most of the studies are focused on reformulating wheat bread, mainly with lentils [62,63], chickpeas [62,64], and peas [62,65]. Protein concentrate and protein isolate from peas, lentils and chickpeas have been successfully incorporated in baked products [62]. However, using concentrated protein leads only to an increase in total protein content, losing the potential health benefits associated with other components present in the flour, including phenolic compounds, fiber, and minerals. 

The incorporation of high amount of pulses has been successfully obtained in biscuits, cake, and other chemically leavened products (see section below). On the contrary, it has been a challenge to make bread, because gluten plays a structuring role in bread. On one hand, pulse proteins are not able to form gluten networks, on the other, weak interactions between pulse and wheat proteins reduce the formation of viscoelastic dough and affect air incorporation and gas retention during leavening, resulting in bread with poor crumb structure and texture. Thus, the addition of chickpea or peas flour is limited to percentages below 10–15% [64]. Generally, pulses are incorporated in common wheat flours, as recently reviewed by Boukid et al. [25]. The differences in observations among studies would most likely be due to differences in types of pulses (lentils, chickpea, etc.) and whether the pulses integrated in the formulation constitute dehulled or hulled flour. Unfortunately, most of the studies did not report any details about the type of flour, i.e., whether they were used after dehulling, making the comparison of the outcomes of different studies difficult. The presence of the structural fiber found in dehulled material would influence dough formation and bread performance. 

Generally speaking, chickpea replacement of less than 10% creates some difficulties in dough preparation, including increased dough stickiness and reduced dough extensibility [66]. When more than 10% of wheat flour is replaced with fiber from peas, lentils, and chickpeas a significant decrease in water absorption is observed, which could be attributed to the higher amount of fiber present [67]. Moreover, the incorporation of flour from dehulled lentils decreases the time required to form the dough and its stability during mixing, together with its resistance to extension, likely due to gluten dilution [68]. Mixtures of wheat and dehulled lentil flours with 20% inclusion have high protein content but low water absorption, resulting in loaves with extremely reduced volumes and dense crumb structures [68]. Thus, high ratio pulse blends are indicated for different baked products such as biscuits (as described in the following section) or extruded products such as noodles. Blends of up to 15% pulse flour generally result in good loaf volume, firmness, and crumb structure. With increasing pulse levels, loaf volume incrementally decreases, and the color of the crumb darkens due to the Maillard reaction [63,66,68], resulting in a decrease in taste and overall acceptability [65]. Specifically, the best sensorial results in terms of appearance, taste, and color are obtained with the addition of up to 10% pulse flour (specifically peas) for bread, whereas higher proportions lead to a worsening of the product’s sensory profile [65]. Finally, the addition of pulses (i.e., chickpeas and peas) has been shown to increase crumb firmness [64,69], likely due to their high amylose content compared to cereals, as mentioned above. The effects of adding pulses on dough rheology and bread quality are reviewed in detail by Boukid et al. [25] and Mohammed et al. [66].

### 3.2. Other Products

As regards biscuits, reformulation by adding pulses is not as challenging as for bread, since the formation of a gas-retaining gluten network during leavening and baking is not required. Moreover, the increase in hardness associated with pulse enrichment is not such a concern as in bread and could even be positive in cookies.

Cookies consist mainly of flour, sugar, and fat, and therefore the addition of pulse flour could improve their nutritional profile. Researchers have published several studies that show how it is possible to reformulate cookies with the addition of different types of pulses such as chickpeas (up to 10%) [70], lupins (up to 20%) [70], green lentils (from 25 to 100%) [71], and navy beans (up to 30%) [72]. These studies showed that, in cookies, the protein content increased proportionally with the addition of pulse flour while reducing dough spread. Pulse flour incorporation leads to darker surface color and a proportional increase in the hardness of the product. Using pulses to enrich bakery products is particularly suitable for gluten-free formulations, in fact, gluten does not play a key role in cookie-making. Malcomson et al. [73] showed that adding 20% yellow pea flour to gluten-free raw materials such as rice flour and tapioca starch did not modify the characteristics of cookies in terms of acceptability and texture.

In cake-baking, several types of pulse flours can be used as an ingredient, such as chickpeas [74] or peas [75]. Gomez et al. [75] focused on cake volume and observed a substantial decrease in the sample supplemented with pulse proteins; also, the resultant bubbles were smaller and more uniformly distributed. Firmness increased while springiness and cohesiveness decreased.

Another product that is well suited for reformulation with pulse flour are crackers. Malcomson et al. [73] added 30% of whole green lentil flour to a commercial cracker formulation. His findings show that crackers supplemented with lentil flour results in a protein-rich cracker with twice the total dietary fiber of wheat crackers. Crackers with lentil flour were darker in color, but their crisp texture and peppery flavor were considered acceptable and comparable to the control. Considering the non-essential role of gluten, crackers are suited not only for reformulations but also complete replacement with pulses including chickpea, green, red lentils pinto bean, navy bean, and yellow pea flours. The pulse-based, gluten-free cracker products investigated by Han et al. [76] have proved to be appealing for consumers, thanks to their health benefits. The sensory aspects of this cracker in terms of color, texture and taste were judged positively and were comparable with existing products on the market.

## 4. Main Barriers to the Use of Pulses and Potential Solutions

Using pulses in food formulations presents some challenges that need to be solved, in view of the nutritional benefits related to their consumption. The first difficulty to be faced is the presence of antinutritional factors, mainly phytic acid and tannins, in the seeds [77], which results in bloating and vomiting after ingestion of raw pulse seeds or flour [78,79]. Nevertheless, the anti-nutritional components may be reduced using different methods such as those recently reviewed by Patterson et al. [80]. The oldest and still widely used method to reduce antinutritional compounds consists of soaking, which leads to a reduction in phytate, which transfers to the soaking water [80]. 

Another method, dehulling consists of removing the outer layer of seed, which reduces cooking time, removes some antinutritional compounds (e.g., tannins) and improves protein digestibility [81]. Finally, thermal treatments, including extrusion, significantly decrease the presence of antinutritional compounds by eliminating heat-labile antinutrients [82]. Besides antinutritional factors, the sensory profile of pulses—i.e., their beany or bitter flavor profile—which depends on the type of pulses, greatly decrease their acceptability and thus their consumption. Traditionally, fermentation and germination have been used to enhance both the nutritional and sensory profiles of pulses, thanks to the production of aroma compounds and sugars [4].

Finally, incorporating pulses in cereal-based products causes important technological issues. In the case of bread, quality is related to an optimum balance of rheologically important gluten-forming proteins (i.e., gliadins and glutenins) and the addition of pulse flour to the wheat flour matrix leads to variations that inevitably worsen bread quality. The presence of pulse proteins not only dilutes gluten but also causes competition between wheat (gliadin and glutenin) and pulse (albumin and glogulin) proteins. Specifically, pulse proteins have a greater number of hydroxyl groups and for this reason they have a higher capacity for water binding [83]. Pulse fiber has also been reported to compromise gluten–gliadin strand formation [67,84]. Two main approaches can be taken to enhance the quality of final products. One depends on choosing suitable ingredients. The second approach consists of applying (bio)-technological treatments to the raw material.

### 4.1. Ingredients

Bread quality can be reestablished by using other ingredients, including vital gluten [68], hydrocolloids [64] or emulsifiers [85]. The fortification of flour with the addition of vital wheat gluten (0.1 g/gram flour) improves its rheology profile compared to that of the control blend, by increasing dough mixing stability, extensibility, and resistance to extension [68]. In particular, adding gluten to wheat–lentil composites significantly increases loaf volume for blends with <40% lentil concentration. Specifically, the concentration of gluten used by Portman et al. [68] could recover the possible loss of loaf volume caused by the addition of 5–15% lentil flour. 

The combination of gluten (5%) and carboxymethylcellulose (5%) was effective in restoring or even improving the quality profile of breads formulated at maximum substitution levels of chickpea (20%), green pea (20%) and soybean (14%) flours [64]. 

Emulsifiers and pectin also significantly improved dough rheology, as well as the nutritional and sensory attributes of pulse-enriched bread [85]. The addition of emulsifiers (up to 1%) in a chickpea-enriched wheat bread significantly increased bread volume, while decreasing crumb firmness. The addition of emulsifiers can help to strengthen the gluten network, which in turn allows for greater pore size expansion resulting in a more porous bread crumb [69].

However, such ingredients in baked products may increase costs and might not satisfy consumer demand for clean label products. As an alternative, using a strong wheat flour could partially compensate for gluten dilution due to pulses. As mentioned above, pulses are generally incorporated in common wheat flours and the maximum enrichment level is 10%. However, when durum wheat semolina is used, the maximum enrichment level for yellow pea flour could go as high as 20%, producing a bread that was more appreciated and more similar to the control [86]. However, the decrease in volume and crumb porosity at 20–30% enrichment level might be counterbalanced by higher dietary fiber and lower glycemic index of the breads [86].

### 4.2. (Bio)-Technological Treatments

Besides the use of suitable ingredients, some biotechnological approaches—including air classification, fermentation, and germination—seem to be effective for enhancing the technological properties of pulses, their fractions, flours and/or enriched products. Table 3 summarizes the main results of the most recent scientific efforts in this field. Conflicting results among studies might be due to differences in plant species as well as variations in processing conditions.

### 4.3. Air Classification

As mentioned before, pulses are an interesting source of proteins and other nutrients. As regards protein isolation, applying wet fractionation under alkaline or acidic conditions yields relatively pure protein isolate, up to and sometimes exceeding 90%. However, the processing conditions (in terms of temperature and pH) are sometimes responsible for protein denaturation and loss of functionality. Moreover, wet extraction impacts negatively on the environment due to the amount of water and energy required. Consequently, dry separation processes are nowadays the preferred methods to separate plant proteins while maintaining their functionality. In this context, air classification has been widely studied, and its basic principles, together with recent applications have recently been reviewed [103,104]. Briefly, this technique exploits the centrifugal and gravitational forces induced by air to separate flour into fine and coarse particles differing in size and density. As a result, flours are separated into fractions characterized by different composition [105]. Feed particles must be sufficiently small and disaggregated in order for air classification to fractionate cell components [105]. For this reason, the efficiency of the separation is enhanced by the size reduction of the flour (by milling) prior to air classification. In addition, in starch-rich legumes, such as peas, starch granules (±20 μm) are embedded in a matrix of protein bodies (1–3 μm), which is fragmented during milling into particles smaller than the starch granules. Then, starch and proteins are easily separated based on size and/or density by air classification. Further information on milling of leguminous commodities has been recently reviewed by Thakur et al. [20]. Very fine milling is not optimal, because starch and fiber particles should be larger than the protein bodies [106]. On the contrary, coarse milling was found optimal to release protein bodies from their matrix, while further milling compromised the purity of the fine fraction [89,107]. 

Numerous studies dealt with the use of air classification up until the 1990s. To the best of our knowledge, most of these studies focused on the functional and nutritional features of these fractions, whereas studies about the incorporation of these flours in baked-products are scarce or null. More recently, Gómez et al. [75] highlighted the positive effect of using the starchy fraction from pea flour on the specific volume of sponge cakes. On the other hand, a worsening of the overall quality of the product was observed when fractions with high protein content were incorporated in the place of starch, which plays a structural role in cakes.

### 4.4. Fermentation

Sourdough fermentation, one of the oldest food biotechnologies, has been widely studied and recently rediscovered for its positive effects on the sensory, textural and nutritional features of baked products. Such changes are related to several biochemical modifications including acidification, proteolysis, activation of several enzymes, and synthesis of metabolites, which affect both dough and bread functionality [108]. More recently, sourdough fermentation has been proposed as a pre-treatment to stabilize or to increase the functional value of wheat-milling by-products [109,110,111]. Moreover, Gobbetti et al. [112] summarizes the most relevant effects of sourdough fermentation on legume flours, in terms of decreases in antinutritional factors (e.g., phytic acid concentration and trypsin inhibitory activity), and raffinose family oligosaccharides. On the other hand, fermentation promotes an increase in free amino acids, gamma-aminobutyric acid, soluble fibers, total phenol concentrations. Although several studies have focused on the nutritional benefits of pulse fermentation, few of them have investigated the effects of sourdough fermentation on dough rheology and the bread-making performance of pulse-enriched flours. Thus, further studies should be focused on such aspects in view of the potential use of sourdough fermentation as an interesting strategy to improve the sensory quality of pulse-enriched breads. Indeed, the sour flavor, which is typical of fermentation with lactic acid bacteria, might hide the off flavor of pulses and thus gain consumer acceptance of pulse-enriched breads [113]. 

### 4.5. Germination

Germination (also known as sprouting) is a re-emerging trend in healthy foods, thanks to its role in improving taste and nutritional properties. Germination is an easy process that traditionally takes place at home. It consists of soaking grains in water until they reach the moisture content necessary to start the growth of seedlings. After the soaking water is drained, the seeds are left to germinate. The germinated grains are then consumed in the form of sprouts or further processed (i.e., dried or roasted). Challenges regarding safety and reproducibility of process, must be solved for industrial applications and to guarantee a safe product with consistent features. Monitoring the process seems the only way to obtain a sprouted product with improved nutritional and sensory properties while maintaining flour performance, which ensures consistent product functionality [114].

Sprouting has been applied for millennia to pulses to reduce their anti-nutritional components, such as trypsin inhibitors and phytic acid. At the same time, enzymes produced during sprouting are also able to degrade ROFs (raffinose family of oligosaccharides) into shorter carbohydrates, eliminating some typical problems associated with their ingestion while developing sweet taste notes in germinated pulses. Moreover, sprouting influences the sensory properties of grains, giving them a typical flavor and odor generally perceived as pleasant. 

The unique flavor profile of sprouted grains is due to the activation of endogenous amylolytic enzymes that transform complex starch molecules into simple oligosaccharides and sugars, which add natural sweetness to products. Thus, the sweetness of grain foods can be enhanced naturally by using the germination process. Moreover, during sprouting, reducing sugars and amino acids are released, which subsequently react during heating, giving rise to Maillard reaction products [115]. Finally, both germination and drying decrease musty and earthy odor notes, favoring the perception of roasted, nutty and intense flavor notes [116] and masking the unpleasant beany flavor in extruded products from soybean [117].

In addition to the nutritional and sensory aspects listed above, germination affects the technological performance of grains and related flours. Surprisingly, germination facilitates the dehulling process for brown chickpeas, mung peas, and pigeon peas [118]. Moreover, the process can influence the cooking properties of pulses by decreasing cooking times and reducing the amount of dispersed solids [119]. The decrease in cooking time for germinated grains is of great interest, since it would facilitate the preparation, and thus the consumption, of whole grains. Indeed, even while reducing the risk of cardiovascular disease and inflammation, the eating of grains is not so common in many countries, due to their long cooking times and bitter and pungent flavor notes [116]. 

The degree of germination-related effects greatly depends on the processing conditions used (i.e., time, temperature and relative humidity), since the biochemical events occurring during germination influence the quality of the ingredients. Recently, controlled germination has been carried out on peas and chickpeas [120]. The applied conditions (3 days, 22 °C and 90% relative humidity) induced mild structural modifications, sufficient to reduce anti-nutritional factors (e.g., phytic acid), without negatively affecting the nutritional quality of the grains (e.g., starch digestibility) [120]. The resulting flour has been proposed as an interesting ingredient for formulating enriched products [101]. Indeed, the reticulating ability of proteins improved as a result of sprouting, and the resultant starch did not interfere with dough development in formulations enriched with nutritionally significant levels of chickpea flour (i.e., 20%) [101]. Using germinated yellow peas in processed foods other than bread—such as white layer cakes, and extruded snacks—also resulted in end products with acceptable characteristics [121].

## 5. Conclusions

Today, consumers are more conscious about the environmental effects and nutritional benefits of foods. In this context, thanks to their nutritional and health-promoting properties, together with their low environmental impact, pulses can be considered a suitable raw material for food production. However, some factors limit their use on an industrial scale. Beside the presence of antinutritional factors, the sensory profile of pulses—i.e., their beany or bitter flavor profile—greatly decreases their acceptability. Traditionally, fermentation and germination have been used to enhance both the nutritional and sensory profiles of pulses, thanks to the production of aroma compounds and sugars. From a technological standpoint, incorporating pulses in cereal-based products is challenging due to the presence of fiber and non-gluten proteins. In wheat-based formulations, not only is gluten diluted by the presence of pulses proteins, but also wheat and pulse proteins compete for water and pulse proteins compete as rival water-binders. Two main approaches can be taken to enhance the quality of end-products. One is based on choosing suitable ingredients, such as strong wheat flour, vital gluten, hydrocolloids or emulsifiers. The second approach consists of the application of (bio)-technological treatments to the raw material, such as air classification, sourdough fermentation, and germination. 

However, most studies seem to adopt an empiric approach from knowledge acquired from cereals, mainly varying ingredients and processing conditions rather than understanding the macromolecule organization associated with good or poor performance. Processes have the ability to modify the characteristics of macromolecules (mainly protein and starch) and their interactions. The extent of these changes is not only defined by the type of process but also by its intensity. In this context, efforts should be devoted to understanding the relation between types of pulses, extent of processes, biopolymer interactions, and product quality in terms of sensory, textural, and nutritional features. As regards the effects of processing on the nutritional characteristics of pulses, most studies often neglect the impact on dough rheology and bread quality. Thus, a multidisciplinary approach is recommended in order to provide solutions/strategies to satisfy both nutritional and technological demands.

## Figures and Tables

**Table 1:** Topics of the main reviews published on pulses (source: Food Science and Technology Abstracts; 2018–2019; updated to 03 July 2019).

Research Area	Topic	Reference
Plant science/agronomy	Breeding	Pratap et al. [7]; Morris et al. [8]; Warsam et al. [9]
	Cultivation	Farooq et al. [10]
Nutrition/Health	Health benefits	Luna-Vita et al. [11]; Harouna et al. [12]; Harsha et al. [13]
	Bioactive compounds	Awika et al. [14]; Chhikara et al. [15]; Yi-Shen et al. [16]
	Allergens	Cabanillas et al. [17]
	Anti-nutritional factors	Avilés-Gaxiola et al. [18]
	Starch digestibility	Jeong et al. [19]
Processing	Milling	Thakur et al. [20]; Scanlon et al. [21]; Vishwakarma et al. [22]
	Enhancing nutritional properties	Van-der-Poe et al. [23]; Nkhata et al. [24]
	Bread fortification	Boukid et al. [25]; Rehman et al. [26]; Zhong et al. [27]
Functionality	General	Foschia et al. [28]; Jarpa-Parra [29]
	Emulsifiers	Burger et al. [30]; Sharif et al. [31]
	Structure-function relationship	Shevkani et al. [32]; Lam et al. [33]

**Table 2:** Potential health benefits of pulses and their mechanisms.

Health Benefits	Key Component	Mechanism	Reference
Colon cancer	Fiber	Anti-proliferative activity and inducing apoptosis in colon cancer cells	Mathers et al. [58]
Heart disease	Fiber	Reduction of blood pressure	Jayalath et al. [57]
	Mono- and polyunsaturated fat; sterols	Increase in high-density lipoprotein (HDL) cholesterol and decrease in both low-density lipoprotein (LDL) and total cholesterol	Bazzano et al. [55]
Diabetes	Resistant starch	Improvement of glucose tolerance as well as insulin sensitivity	Jenkins et al. [56]
Weight Control	Fiber	Interference with caloric intake by increasing chewing time and satiety	McCrory et al. [53]

**Table 3:** Aim and main results of the most recent studies on the (bio)-technological approaches applied to pulses.

Air Classification			
Reference	Type of Pulses	Aim	Outcome
Rempel et al. [87]	pea	To assess the effects of milling and air classification on chemical composition.	Production of fine fractions having 90% of particles diameters smaller than 22 µm and high in protein (85–87%), fat (74–95%), and minerals (66–76%).
Simons et al. [88]	pinto bean	To produce high-starch fractions and assess their potential applications.	Production of protein (yield: 20%, size: ≤15 µm) and starch (yield: 80%, size: 15–45 µm) fractions. The latter characterized by high viscosity and high resistant starch that might be used in food formulations to lower the glycemic index and/or increase viscosity of foods.
Pelgrom et al. [89]	pea, lupine	To assess the effects of processing on the effectiveness of air classification.	Hydration, de-hulling or defatting prior to air classification were found effective in increasing protein yield and content.
Coda et al. [90]	faba bean	To enhance flour functionality by using fractions obtained by air classification.	Production of a starch-rich fraction with a low content in antinutritional factors.
Pelgrom et al. [91]	pea, bean, chickpea, lentil	To optimize the separation of starch granules from cell wall fibers and protein bodies.	Optimization of separation when the particle size distribution of flour overlaps with that of isolated starch granules.
Gómez et al. [75]	pea	To assess starch fraction suitability in cake making.	Using starch concentrate fraction did not affect negatively on cake quality but was found unacceptable for consumers. On the other hand, using protein fraction negatively affects cake quality.
**Fermentation**			
**Reference**	**Type of legumes**	**Aim**	**Outcome**
Coda et al. [92]	faba bean	To investigate the effects of pulse sourdough on bread quality.	Using pulse sourdough positively affects the amino acid profile, protein digestibility, protein biological value, and glycemic index of bread.
Xu et al. [93]	faba bean	To assess the potential of different lactic acid bacteria in the production of exopolysaccharides and their impact on product texture.	*Ln. pseudomesenteroides* DSM 20193 showed the highest potential in the production of exopolysaccharides and texture modification in the related dough.
Rizzello et al. [94]	faba bean	To assess the effects of fermentation on of the pyrimidine glycoside vicine and convicine.	48 h of incubation with *L. plantarum* led to the degradation of the pyrimidine glycosides and aglycone derivatives.
Curiel et al. [95]	nineteen traditional Italian legumes	To assess the effects of sourdough fermentation on the functional and nutritional characteristics of pulses.	Fermentation promoted an increase in free amino acids, soluble fibers, and total phenols. Raffinose and condensed tannins decreased, while the level of gamma-aminobutyric acid, antioxidant and phytase activities markedly increased.
Rizzello et al. [96]	nineteen traditional Italian legumes	To investigate the effects of fermentation on the concentration of lunasin-like polypeptides.	Sourdough fermentation increased the amount of lunasin-like polypeptides, due to proteolysis of the native proteins. A marked inhibitory effect on the proliferation of Caco-2 cells was also observed.
Coda et al. [90]	faba bean	To assess the effects of air classification and lactic acid bacteria fermentation on the decrease in anti-nutritional factors and starch and protein digestibility of pulses.	The combination of air classification and fermentation was effective in decreasing/removing the anti-nutritional factors as well as improving the free amino acid content and protein digestibility.
Rizzello et al. [97]	chickpea, lentil, bean	To evaluate the effects of fermentation on nutritional, sensory and functional characteristics of pulse-enriched bread.	*L. plantarum* was the dominant lactic acid bacteria species in the wheat–legume sourdough. Using sourdough maximized the nutritional (by increasing the essential free amino acids, phenols and dietary fiber, and decreasing the hydrolysis index), sensory and functional properties of pulse-enriched bread.
**Germination (or Sprouting)**			
**Reference**	**Type of legumes**	**Aim**	**Outcome**
Ouazib et al. [98]	chickpea	To investigate the impact of germination on rheological and bread-making performance of pulses.	Changes in starch upon germination significantly affected the rheological properties of the related flour. Germination negatively affected the overall acceptability of bread.
Ertaş [99]	lupin	To study the effects of sprouting on the physical and chemical properties of pulses and their bread-making performance	Sprouting of pulses enhanced the technological (volume, specific volume, symmetry and texture) and nutritional properties of bread.
Mondor et al. [100]	pea	To assess the effect of malted peas (10%) on bread quality.	The malting process did not affect the mixing property of the dough.
Marengo et al. [101]	chickpea	To assess the impact of sprouting on macromolecular and micronutrient profiles and rheological properties of chickpeas and chickpea flour–enriched dough (wheat/chickpea ratio = 100:20)	Sprouting enhanced the reticulating ability of proteins. Starch changes upon sprouting did not interfere with dough mixing properties and improved its leavening properties. Thus, sprouting of pulses might provide a good opportunity for developing new products with increased nutritional value.
Montemurro et al. [102]	chickpea	To investigate the effects of germination and sourdough fermentation on grain quality	Combining fermentation with sprouting further enhanced the nutritional and functional characteristics of flours, through the release of peptides and free amino acids, phenolic compounds and soluble fibers, and the decrease in several antinutritional factors. Bread enriched in fermented sprouted flour showed peculiar sensory profiles, and high protein digestibility and low starch availability, compared to the control sample.
